# Neurodevelopmental and behavioral consequences of perinatal exposure to the HIV drug efavirenz in a rodent model

**DOI:** 10.1038/s41398-019-0420-y

**Published:** 2019-02-11

**Authors:** Lisa van de Wijer, Lidiane P. Garcia, Sabrina I. Hanswijk, Juliette Rando, Anthonieke Middelman, Rob ter Heine, Quirijn de Mast, Gerard J. M. Martens, André J. A. M. van der Ven, Sharon M. Kolk, Arnt F. A. Schellekens, Judith R. Homberg

**Affiliations:** 1 Department of General Internal Medicine, Radboud University Medical Center, Nijmegen, The Netherlands; 2 Department of Molecular Animal Physiology, Donders Institute for Brain, Cognition, and Behaviour, Centre for Neuroscience, Radboud University, Nijmegen, The Netherlands; 3 Department of Cognitive Neuroscience, Donders Institute for Brain, Cognition, and Behaviour, Centre for Neuroscience, Radboud University Medical Center, Nijmegen, The Netherlands; 4 Department of Pharmacy, Radboud University Medical Center, Nijmegen, The Netherlands; 5 Department of Molecular Neurobiology, Donders Institute for Brain, Cognition, and Behaviour, Centre for Neuroscience, Radboud University, Nijmegen, The Netherlands; 6 Department of Psychiatry, Donders Institute for Brain, Cognition, and Behaviour, Centre for Neuroscience, Radboud University Medical Center, Nijmegen, The Netherlands

## Abstract

Efavirenz is recommended as a preferred first-line drug for women of childbearing potential living with human immunodeficiency virus. Efavirenz is known for its central nervous system side effects, which are partly mediated by serotonergic actions. The neurotransmitter serotonin exerts neurotrophic effects during neurodevelopment and antenatal exposure to serotonergic agents has been linked to developmental delay. Although the teratogenic risks of efavirenz appear to be minimal, data on long-term developmental effects remain scarce. Here, we aimed to investigate the short- and long-term behavioral and neurodevelopmental effects of perinatal efavirenz exposure. We treated pregnant rats from gestation day 1 until postnatal day 7 with efavirenz (100 mg/kg) or vehicle. We measured behavioral outcomes in male offspring during the first 3 postnatal weeks, adolescence and adulthood, and conducted brain immunohistochemistry analyses after sacrifice. Perinatal efavirenz exposure resulted in reduced body weight and delayed reflex and motor development. During adulthood, we observed a decrease in the total number of cells and mature neurons in the motor cortex, as well as an increase in the number of Caspase-3-positive cells and serotonergic fibers. Together, our data show a developmental delay and persistent changes in the brain motor cortex of rats exposed to efavirenz perinatally. Because over 1 million children born annually are exposed to antiretroviral therapy, our findings underline the need for clinical studies on long-term neurodevelopmental outcomes of perinatal exposure to efavirenz.

## Introduction

Every year, an estimated 1.4 million women living with human immunodeficiency virus (HIV) become pregnant. The use of antiretroviral therapy (ART) during pregnancy, delivery, and breastfeeding successfully reduces the risk of mother-to-child transmission of HIV to <5%^1^. The latest interim guidelines of the World Health Organization (WHO) recommend dolutegravir as the general drug of choice for people living with HIV^2^. However, due to concerns about neural tube defects among first-trimester dolutegravir exposures, efavirenz (EFV) remains the preferred option in women of childbearing potential during the periconception period^3^. Research on the safety of EFV during pregnancy has focused largely on infant health shortly after birth^4^. Although risks for gross teratogenicity seem to be minimal, research on long-term neurodevelopmental effects of perinatal exposure to EFV remains scarce^5,6^.

EFV passes through the placenta and is present in breast milk, resulting in detectable concentrations in the blood of fetuses and breast-fed infants^7,8^. After entry into the blood stream, both EFV and its primary metabolite 8-hydroxy-efavirenz (8-OH-EFV) readily penetrate the cerebrospinal fluid and target various cellular pathways within the central nervous system (CNS), predominantly the serotonergic system^9–12^. For example, EFV acts as a serotonin(5-HT)6 receptor inverse agonist, 5-HT2A, 5-HT2C, and 5-HT3A receptor antagonist, and a blocker of the 5-HT transporter (5-HTT)^13^. In rats, EFV preferentially binds to the 5-HT2A receptor^14^.

Importantly, 5-HT exerts neurotrophic functions during early development^15,16^. Increases in brain 5-HT levels, induced by genetic 5-HTT inactivation, have been shown to alter the serotonergic innervation of the prefrontal cortex^17^, migration of inhibitory neurons to the neocortex^18^, and maturation of the sensory cortex^19^. The latter has also been observed after pharmacological 5-HTT inhibition by prenatal selective serotonin reuptake inhibitor (SSRI) exposure^18,20^. Both genetic and pharmacological 5-HTT modulation during early development have been associated with a delay in reflex and motor development, disturbed sensorimotor gating, decreased social behavior, and anxiety and depression-like phenotypes^21–25^. Moreover, children perinatally exposed to SSRIs show reduced language and motor development, and a twofold increased risk of autism spectrum phenotypes^26–28^. Given that EFV particularly targets the serotonergic system, we hypothesized that perinatal EFV exposure might also lead to long-lasting neurodevelopmental consequences.

Here, we aimed to investigate the short- and long-term behavioral and neurodevelopmental effects of perinatal EFV exposure in a rodent model. We conducted a behavioral test battery including tests for reflex development, motor performance, sensorimotor gating and anxiety- and depressive-like behavior, during early life, adolescence, and adulthood. Throughout the treatment period, we monitored maternal care. Because we observed changes in motor behavior, we investigated the cytoarchitecture of the motor cortex to study the underlying cellular mechanisms. Our results indicate that perinatal EFV exposure is associated with neurodevelopmental delay, accompanied by long-lasting changes in motor cortex morphology.

## Materials and methods

### Animals

Rats used in this experiment were bred in-house from Wistar male breeders and nulliparous Wistar females weighing 185–215 g, purchased from Charles River, Cologne, Germany. After a 2-week acclimatization period, female rats were subjected to a timed mating procedure (using Impedance Checker MK-10B, Muromachi Kikai, Tokyo, Japan) as described previously^25^. Pregnancy was determined by observation of a vaginal plug the day after breeding gestational day (GD) 1. Pregnant rats were alternately assigned to daily treatment with EFV or vehicle from GD1 to postnatal day (PND) 7 by order of birth. PND7 resembles the human functional brain maturity around birth^29^. Litters were culled to 10 pups and pups were weaned on PND21. One EFV-exposed pup died at PND17 (cause unknown) and was excluded from analysis. Male offspring from four EFV-exposed (*n* = 24) and four vehicle-exposed nests (*n* = 20) were used for experiments. Developmental milestones and behavior were assessed during the first 3 weeks of life (PND2–21), adolescence (PND34–35), and adulthood (PND69–70). After completion of the last experiments, rats (PND73–75) were sacrificed. Animal numbers used for each test are reported in the respective figure panels, where appropriate, and in Supplementary Table 1.

**Fig. 1:**
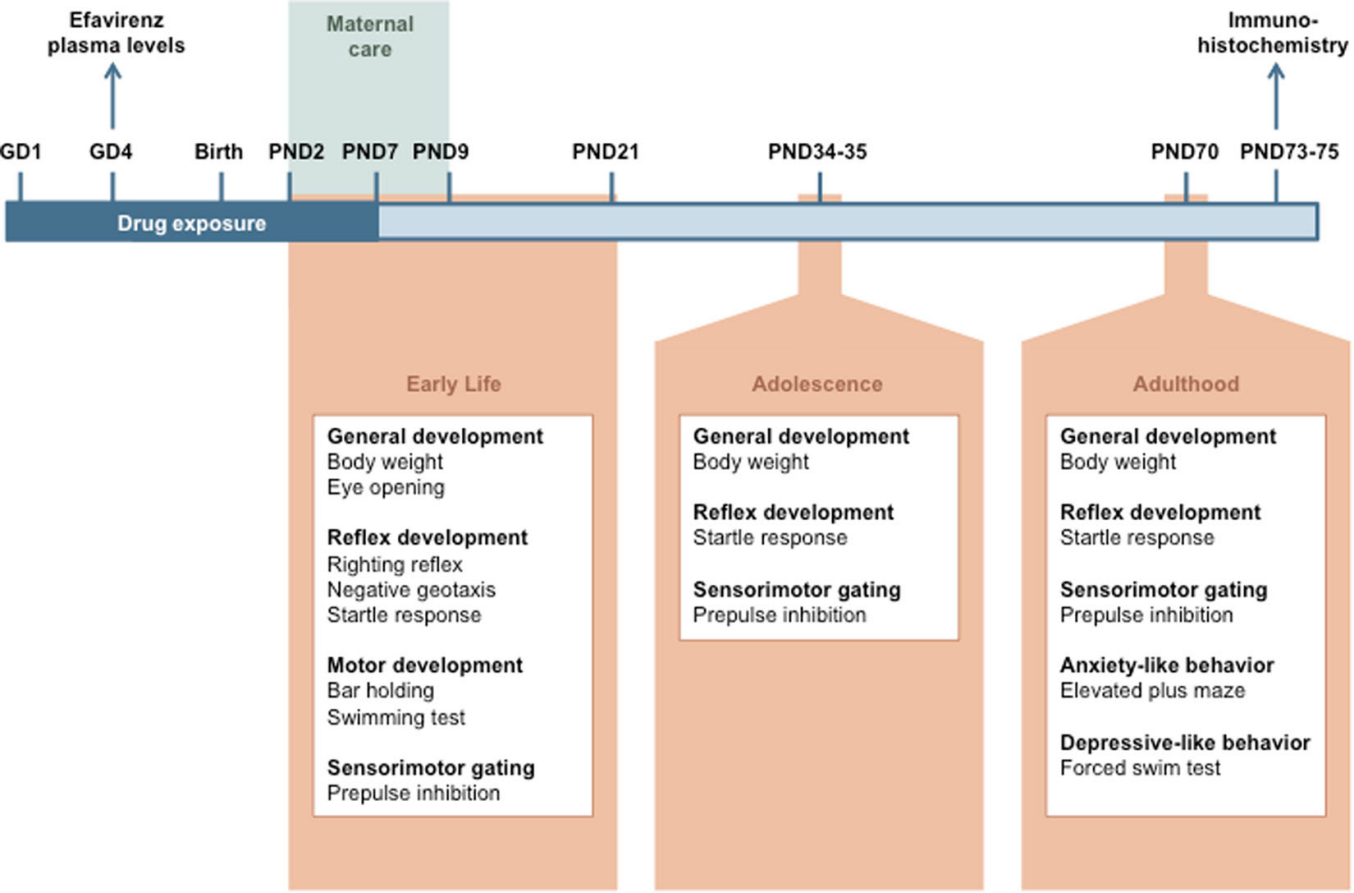
Experimental timeline. Pregnant dams were treated with efavirenz (EFV) (*n* = 4) or vehicle (*n* = 4) from GD1 until PND7. At GD4, plasma levels of EFV were determined 1.5 h post administration. Developmental milestones and behavior were assessed during the first 3 weeks of life (PND2–21), adolescence (PND34–35), and adulthood (PND69–70). Maternal care was monitored from PND2 until PND9. After completion of the last experiments, rats (PND73–75) were sacrificed and their brains were removed for immunohistochemistry analyses. GD gestational day, PND postnatal day

Figure 1 shows a schematic representation of the experimental timeline.

Sample sizes were based on power calculations from previous studies using comparable animal models and similar behavioral or molecular analyses^23–25,30^. Animals were tested randomly and both investigators and caregivers were blinded to the groups during experiments and outcome assessment. Blinding was ensured using coded treatment containers of identical appearance. Videos of the reflex and motor performance tests were re-analyzed by a second blinded researcher resulting in similar results.

Animals were housed in pairs in standard Macrolon® type 3 cages in temperature-controlled rooms (21 °C ± 1 °C) under a standard 12-h light/dark cycle (lights on at 07:00 hours) with food and water available ad libitum (Sniff, long cut pellet, Bio Services, Uden, The Netherlands). Experiments were carried out in accordance with the European Communities Council Directive (2010/63/EU) and approved by the Committee for Animal Experiments of the Radboud university medical center Nijmegen, The Netherlands (ref no. 2012-236).

### Drug treatment

EFV or vehicle was administered blindly by oral gavage in a volume of 5 mL/kg. We used a dose of 100 mg/kg, based on previous work demonstrating plasma levels within the human therapeutic range (1.0–4.0 mg/L)^31^ and unpublished pilot work. Drug solution was prepared by diluting EFV oral suspension (Stocrin suspension 30 mg/mL, Merck Sharp & Dohme, Haarlem, The Netherlands) with distilled water. As vehicle, we used a 1% cellulose suspension (Genfarma B.V., Maarssen, The Netherlands), enriched with the EFV solution additives, consisting of medium chain triglyceride oil (Newpharma, Liège, Belgium) and strawberry and peppermint essence (Lecocq N.V./S.A., Zonhoven, Belgium).

### Measurement of EFV in blood plasma

Ninety minutes after drug administration at GD4, we collected blood, obtained by tail cut, of all pregnant rats (*n* = 8). Blood was collected in Microvette CB 300 tubes (containing EDTA; Sarstedt, Germany) and centrifuged for 15 min at 4 °C with a speed of 4000 rpm. The supernatant (plasma) was stored at −20 °C until analysis. Supernatants from the experimental group were used for determination of plasma peak EFV levels using a validated reversed phase ultra-performance liquid chromatography with ultraviolet detection bioanalytical assay, validated for human plasma. For the current analysis, quality control samples prepared with blank rat plasma were included to assure validity of the assay. During the bioanalysis, the quality control samples in rat plasma did not deviate more than 15% from the theoretical value.

### Maternal care

Maternal care was scored from PND2 to PND9 using a procedure adapted from Ivy et al.^32^. Litters were observed during three sessions per day of 75 min each: at 7:30 a.m., 1:30 p.m. (light phase), and 7:30 p.m. (dark phase). Throughout each session, behavior was scored every third minute (25 observations per session). The behaviors scored include: (1) nursing more than half of the nest, (2) licking or grooming any pup, (3) spending time out of the nest, (4) self-licking or grooming, and (5) eating or drinking.

### General development

Body weight was measured daily during early life (PND2–21), adolescence (PND34), and adulthood (PND70). In addition, we recorded eye opening every morning from PND13 until both eyes were open. Scores were defined as: (0) both eyes closed, (1) one eye open, (2) both eyes open. An eye was considered “open” if the palpebral fissure was ≥ 2 mm^33^.

### Behavioral development

The experimental test battery was adapted from Kroeze et al. and included the following functional domains:^25^ reflex development (righting reflex, negative geotaxis, and acoustic startle reflex), motor development (swimming performance and bar holding), sensorimotor gating (prepulse inhibition [PPI]), and anxiety- and depressive-like behavior (elevated plus maze and forced swim test).

#### Reflex development—righting reflex, negative geotaxis, acoustic startle reflex

During the righting reflex test, rats (PND2–10) were placed on their back, while recording the time until they returned to prostate position, with a maximum of 60 s^34^. Negative geotaxis was examined using a 40° inclined wooden plank with a wire mesh. Rats (PND4–14) were placed facing down the slope and allowed to turn 180° within 90 s^21^. All test sessions were recorded for later rescoring by a second researcher. See below for methods of the acoustic startle reflex.

#### Motor development—swimming performance, bar holding

During the swimming performance test, rats (PND8, 10, 12, 14, 22) were dropped from ± 20 cm into a basin filled with 27 °C water and observed for 5–10 s. Scoring was based on the position of the nose in the water: (1) entire head underwater, (2) nose underwater, ears partially underwater with the back of head above water level, (3) nose above water level but ears partially underwater, (4) entire head above water level^35^. Bar holding was tested by positioning rats (PND10–21) with their forepaws on a wooden bar (3 mm × 40 cm), suspended 45 cm above the bench surface. The latency to fall from the bar was recorded, with a maximum of 50 s. If the rat fell immediately, the procedure was repeated up to three times^25,36^.

#### Acoustic startle reflex and sensorimotor gating—PPI

At PND21, 35, and 70, acoustic startle and PPI experiments were performed in four acoustic startle chambers (San Diego Instruments, San Diego, USA), according to the methods of Sontag et al.^37^. Acoustic startle response was defined as the mean startle amplitude from the 10 test blocks of the startle trials. The percentage of prepulse inhibition was calculated using the formula: 100 – (average of startle amplitude on prepulse trial/average of startle amplitude on startle trial) × 100%^37^.

#### Anxiety-like behavior—elevated plus maze

The elevated plus maze is a polyvinylchloride apparatus with two open (50 × 10 cm, light intensity 12.1 lux) and two enclosed (50 × 10 × 40 cm, light intensity 4.5 lux) arms, elevated at a height of 50 cm^38^. Rats (PND69) were placed in the center of the elevated plus maze facing one of the open arms and allowed to freely explore the maze for 5 min. Movements were measured using the EthoVision XT9 Tracking System, Noldus, Wageningen, The Netherlands.

#### Depressive-like behavior—forced swim test

During the induction phase, rats (PND69) were placed in cylindrical glass tanks (24 cm diameter × 65 cm height filled to 35 cm with 22 °C water) for 15 min. After 24 h, rats (PND70) were placed in the same tank for 5 min (test phase). Immobility was defined as no movements or minimal movements necessary to keep the nose above water level for ≥2 s. While slight movements of forepaws or paw placement on cylinder walls were still considered immobility, active climbing, diving, and swimming were scored as mobility^24^. The duration of immobility (s) was recorded with Observer XT 12.5 (Noldus Information Technology, Wageningen, The Netherlands). Both phases of the forced swim test (induction and test phase) were performed at the end of the testing days (after completion of the other behavioral tests).

### Immunohistochemistry

After a minimum of 2 days after completion of the last behavior experiments, rats (PND73–75) were deeply anesthetized by an intraperitoneal injection of sodium pentobarbital (200 mg/kg) and perfused transcardially with cold phosphate-buffered saline (PBS), followed by 4% paraformaldehyde (PFA). Brains were quickly removed and immersed in 4% PFA for 48 h. Next, brains were washed in PBS, placed in a 30% sucrose solution, frozen on dry-ice, and stored at −80 C. In all, 16 μm sections were cut on a Microm Cryostat, mounted on Superfrost® Plus slides (Thermo Fisher Scientific, Waltham, MA, USA), air-dried and stored desiccated at −20 °C. Cryosections were obtained and stained immunohistochemically as previously described^17^, using the following antibodies: mouse anti-NeuN (1:500, Merck Millipore, Bedford, MA, USA; MAB377), rabbit anti-Cleaved Caspase-3 (Casp3, 1:500, Cell Signaling Technologies, Danvers, MA, USA; ASP175), rabbit anti-5-HT (1:500, Sigma-Aldrich Chemie, Zwijndrecht, The Netherlands; S5545), and rabbit anti-TH (1:500, Merck Millipore, Bedford, MA, USA; AB152), species-specific Alexa-conjugated secondary antibody (1:500, Thermo Fisher Scientific; A32732). After washing in PBS, sections were counterstained with fluorescent 2-(4-Amidinofenyl)-1H-indole-6-carboxamidine (DAPI,1:1000, Thermo Fisher Scientific; 62248) diluted in PBS for 15 min, washed extensively in PBS and embedded in 90% glycerol in PBS. For visualization, a Leica DMRA fluorescence microscope equipped with a DFC340FX camera and LASAF software was used.

### Quantification

For quantification of cells and fibers in coronal sections, pictures of at least five brains per group were acquired similar to Witteveen et al.^39^. Stained cells were counted in radial units of 0.1-mm wide in the presumptive primary motor cortex (M1) of anatomically matched brain sections using Photoshop CS6 (Adobe). The overall cortical length, above white matter, of M1 was divided into 10 equal bins (bin1 within the deep cortical layers and bin10 within the presumptive layer 1) within this rectangle, ImageJ, including the NeuronJ plugin, was used for counts and measurements (National Institutes of Health, Bethesda, USA).

### Statistical analysis

Data are expressed as mean ± standard error of the mean (SEM) unless indicated otherwise. Continuous behavioral data were analyzed using repeated-measures analysis of variance (ANOVA). In case of significant violations of sphericity or error variances, the Greenhouse–Geisser adjustment was applied and *df* were adjusted. Significant ANOVA results were followed by post-hoc independent *T*-tests, in order to identify the specific time points with the largest effect of EFV. Differences in non-continuous data were analyzed by Pearson’s chi-square (or Fisher’s exact). Immunohostochemical data were analyzed using one-way ANOVA. We observed extreme outliers in the acoustic startle/PPI test and removed these according to Tukey’s principles. Cumulative behavioral development was defined using area-under-the-curves (AUC) for continuous data (righting reflex, negative geotaxis, bar holding) and sum scores for non-continuous data (swimming performance). Spearman’s correlations were used to correlate behavioral development with immunohistochemistry results. Statistical analyses were performed using SPSS version 22.0 (SPSS Inc., Chicago, IL, USA) and GraphPad Prism3 software (California Corporation). All tests were performed using a two-tailed hypothesis, with significance set at 0.05. The Benjamini–Hochberg procedure was used for multiple comparisons behavioral *post-hoc* analyses with a false discovery rate (FDR) of 0.1.

## Results

### Pregnancy outcomes and maternal care

**Fig. 2:**
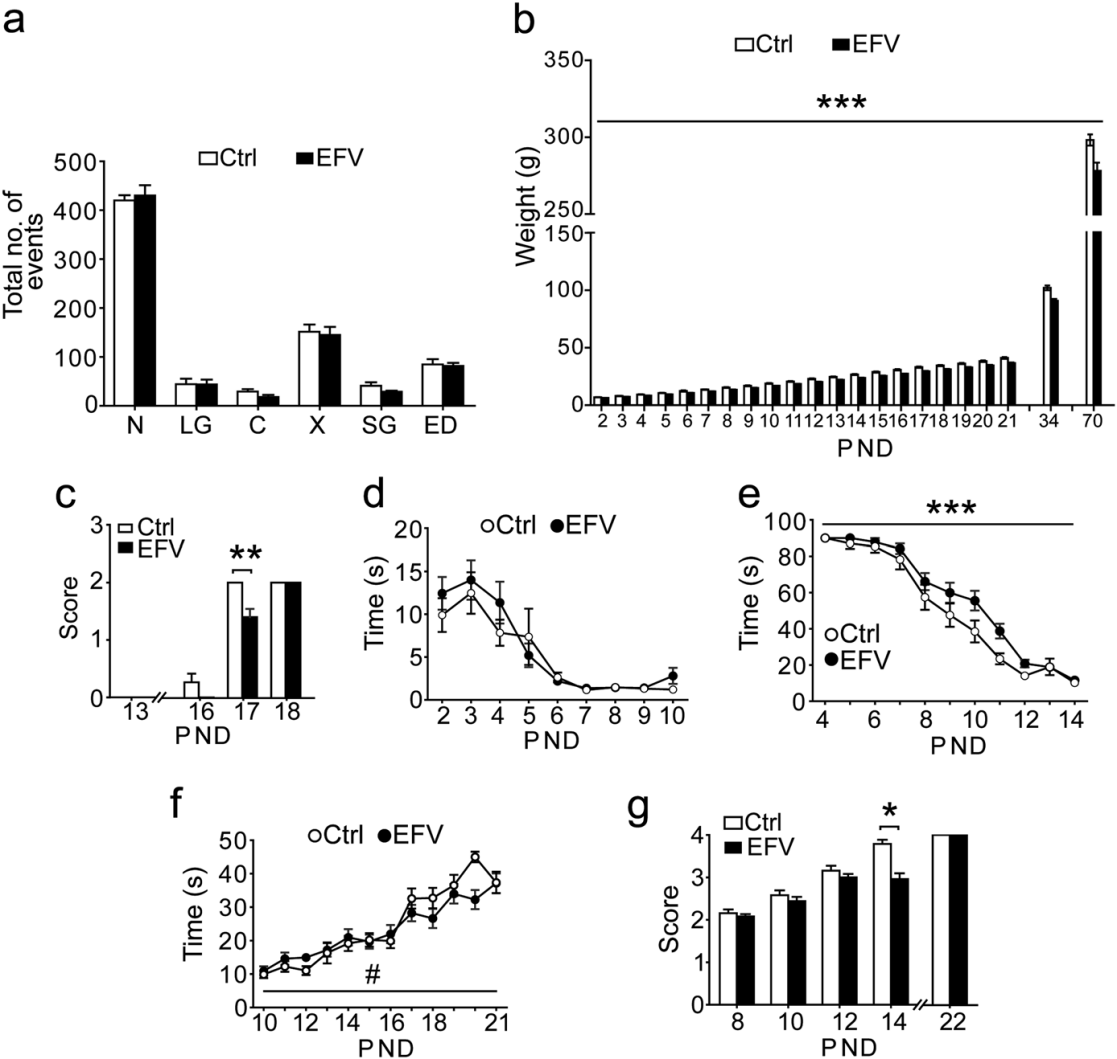
Perinatal exposure to EFV significantly affects body weight, eye opening, reflex and motor development, without effects on maternal care. **a** No effect of EFV on maternal behaviors. (*n* = 4 litters per group, PND2–9). **b**, **c** Effects of perinatal EFV on general development. **b** Main effect of EFV on body weight, with EFV-exposed rats showing reduced body weight. **c** EFV affects eye opening (PND17) scored as (0) both eyes closed; (1) one eye open; (2) two eyes open (**d**, **e**). Effect of perinatal EFV on reflex development. **d** No effect of EFV on righting time (s) in the righting reflex test. **e** Main effect of EFV on negative geotaxis test, with EFV-exposed rats exhibiting longer latencies (s) until turning 180°. **f**, **g** Effects of perinatal EFV on motor development. **f** No main effect of EFV on the latency (s) to fall off the bar in the bar holding test, with a trend in EFV × time interaction. **g** EFV affects swimming performance (PND14), scored as (1) entire head underwater, (2) nose underwater, ears partially underwater with the back of head above water level, (3) nose above water, ears partially underwater, (4) entire head above water (PND8, 10, 12, 14, 22). EFV *n* = 24, Ctrl *n* = 19. Data represent mean ( ± SEM), ^#^*p* < 0.10, *p < 0.05, **p < 0.01, ***p < 0.001, analyzed using repeated-measures ANOVA (**a**, **b**, **d**, **e**, **f**) or Fisher’s exact (**c**, **g**). Ctrl vehicle, EFV efavirenz, PND postnatal day, N nursing, LG licking/grooming, C contact, X out of nest, SG self-grooming, ED eating/drinking, ANOVA analysis of variance

On GD4, 1.5 h following the administration of EFV, mean ( ± SEM) EFV plasma levels in EFV-treated dams (*n* = 4) were 0.28 ± 0.13 mg/l. Gestational length did not differ between EFV (22.0 ± 0.0 days) and controls (22.3 ± 0.2 days, *p* = 0.69). No apparent anatomical abnormalities were seen in the EFV-exposed offspring. Furthermore, there were no significant differences in the number of pups per litter (EFV 10.8 ± 0.7 vs. controls 8.8 ± 1.3, *p* = 0.34), nor differences in the survival rates for pups at weaning. Finally, the mean ( ± SEM) percentage of female pups per litter did not differ between groups (EFV 42 ± 3% vs. controls 41 ± 6%, *p* = 0.89; Supplemental Table S2). To examine effects of EFV on dam–pup interactions, maternal behavior was scored from PND2 until PND9 and did not differ between groups (*p* > 0.10 for main effects of EFV and interaction between time and EFV; Fig. 2a).

### General development

Figure 2b shows the body weights of EFV- and vehicle-exposed rats during early life (PND2–PND21), adolescence (PND34), and adulthood (PND70). Body weight increased with age (*F*_(1.2, 49.3)_ = 6209.51, *p* < 0.0001). Perinatal EFV exposure was associated with reduced weight gain (main effect EFV: *F*_(1, 41)_ = 29.09, *p* < 0.0001; time × EFV interaction effect: *F*_(1.2 ,49.3)_ = 7.99, *p* = 0.0045). Body weight was significantly reduced in EFV-exposed rats for all testing days (post-hoc FDR-adjusted *p*-values *≤* 0.0052). Eye opening was scored daily from PND13–PND18. On PND17, EFV-exposed rats showed a delay in eye opening *(*FDR-adjusted *p* = 0.0027, Fisher’s exact; Fig. 2c).

### Reflex and motor development

**Fig. 3:**
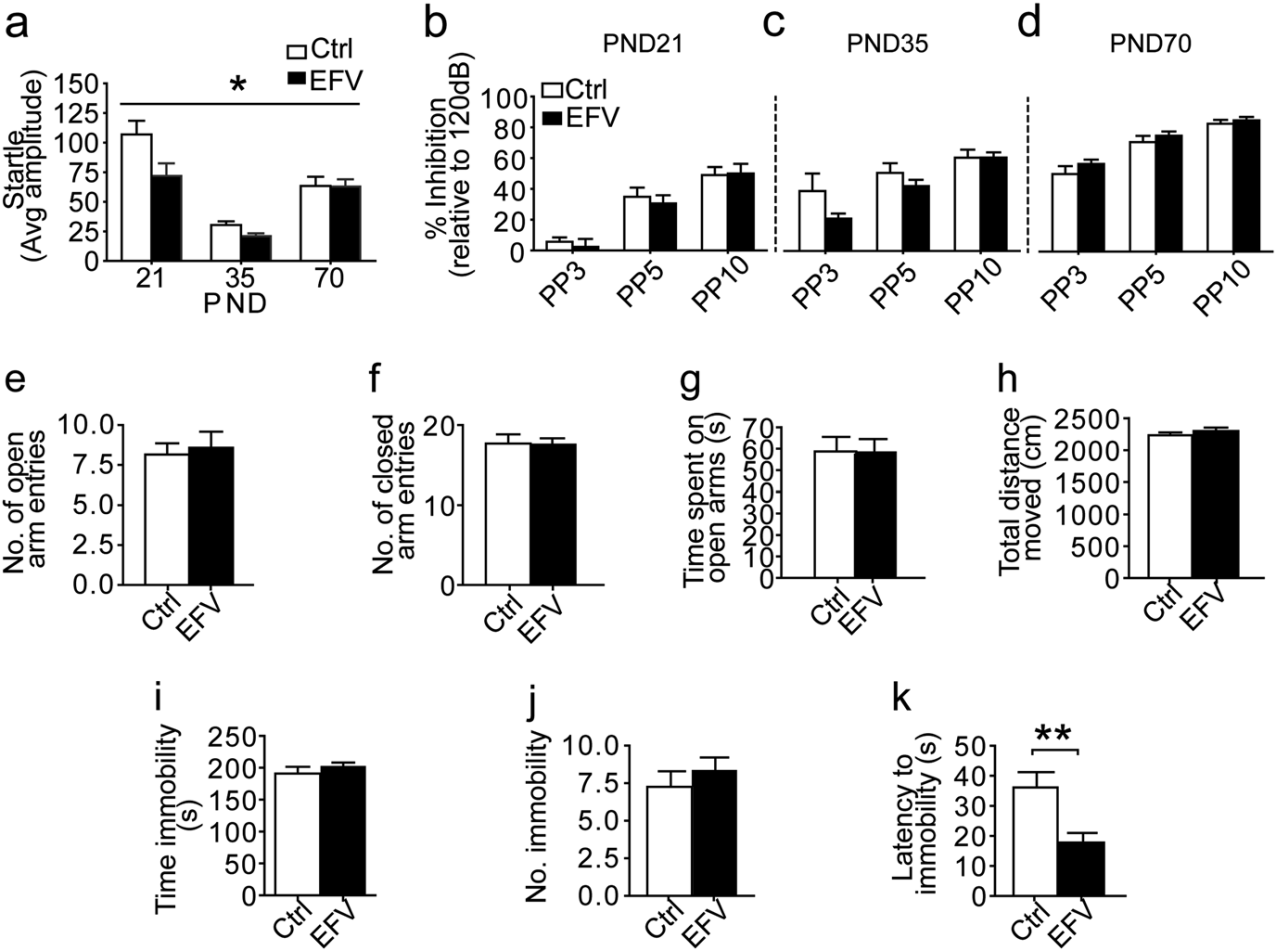
Perinatal EFV exposure affects the startle reflex and latency to immobility in the forced swim test, but has no effects on prepulse inhibition, or anxiety- and depressive-like behavior. **a**–**d** Effect of perinatal EFV on acoustic startle reflexes and prepulse inhibition (PPI). **a** Main effect of EFV on average startle amplitude development, with EFV-exposed rats exhibiting shorter startle amplitudes. No effect of EFV on PPI on **b** PND21, **c** PND35, and **d** PND70. PPI was calculated as: 100–(average of all startle amplitudes on prepulse trial/startle amplitude on startle trial) × 100%. Three different prepulses were tested: 3 dB (PP3), 5 dB (PP5) or 10 dB (PP10) above background noise. **e**–**h** No effect of perinatal EFV on anxiety-like behavior in the elevated plus maze. **e** Number of open arm entries, **f** number of closed arm entries, **g** time (s) spent in open arms, and **h** total distance (cm) moved. **i**–**k** Effects of perinatal EFV on depressive-like behavior in the forced swim test. **i** No effect of EFV on the time (s) spent immobile, or **j** the number of immobility times. **k** Main effect of EFV on the latency (s) to immobility, with EFV-exposed rats exhibiting shorter latencies. EFV *n* = 24 (*n* = 22 for the forced swim test) and Ctrl *n* = 19. Data represent mean ( ± SEM), *p < 0.05, **p < 0.01, analyzed using repeated-measures ANOVA (**a**–**d**) or one-way ANOVA (**e**–**k**). Ctrl vehicle, EFV efavirenz, PND postnatal day, PP prepulse, ANOVA analysis of variance

The time until the rats were able to roll from back to the front (righting reflex) reduced with age (*F*_(3.8, 158.4)_ = 20.09, *p* < 0.0001; Fig. 2d). There were no main or interaction effects of EFV on righting time. In the negative geotaxis test, the time until turning decreased with age (*F*_(5.0, 207.6)_ = 127.04, *p* < 0.0001; Fig. 2e). Rats perinatally exposed to EFV showed a developmental delay for negative geotaxis (main effect *F*_(1, 41)_ = 13.59, *p* = 0.00066; time × EFV interaction *F*_(5.1, 207.6)_ = 0.1.17, *p* = 0.32). Post-hoc analyses showed longer turning times in EFV-exposed animals on PND11 and PND12 (post-hoc FDR-adjusted *p*-values for both days *p* = 0.075). We observed an improvement of performance in the bar holding test over time (*F*_(7.5, 306.6)_ = 35.02, *p* < 0.0001; Fig. 2f). We did not see any main effect of EFV on bar holding (*F*_(1, 41)_ = 1.17, *p* = 0.29), but we observed a trend in time × EFV interaction (*F*_(7.5, 306.6)_ = 1.81, *p* = 0.079), with significant differences between groups on PND20 (post-hoc FDR-adjusted *p* = 0.0048). In the swimming test, EFV-exposed rats scored significantly lower than their controls on PND14 (FDR-adjusted *p* = 0.00046, Fisher’s exact; Fig. 2g). Finally, startle reflexes were assessed. The amplitudes of the startle response increased over time (*F*_(1.5, 57.1)_ = 33.32, *p* < 0.0001; Fig. 3a), with a main effect of EFV (*F*_(1, 37)_ = 5.65, *p* = 0.023), and a trend for time × EFV interaction (*F*_(1.5, 57.1)_ = 2.72, *p* = 0.087). Post-hoc analyses showed significantly lower startle responses in the EFV group in early life (PND21, post-hoc FDR-adjusted *p* = 0.049) and adolescence (PND35, post-hoc FDR-adjusted *p* = 0.049), but not in adulthood (PND70, post-hoc FDR-adjusted *p* = 0.95). Taken together, these data demonstrate that EFV exposure affected early reflex and motor development, which normalized in later life.

### Development of sensorimotor gating and anxiety- and depressive-like behavior

For all prepulses, the effects of PPI increased over time (*F*_(1.7, 56.3)_ = 36.76, *p* < 0.0001 for 3 dB, *F*_(2, 70)_ = 33.21, *p* < 0.0001 for 5 dB, and *F*_(1.18, 42.4)_ = 8.57, *p* = 0.0037 for 10 dB). We did not observe any main or interaction effects of EFV on PPI, regardless of the prepulse strength (Fig. 3b–d). We found no group differences in elevated plus maze open arm time (*F*_(1, 41)_ = 0.01, *p* = 0.92; Fig. 3e). The number of open arm entries (*F*_(1, 41)_ = 0.11, *p* = 0.74) and closed arm entries (*F*_(1, 41)_ = 0.01, *p* = 0.92) also did not differ between control- and EFV-exposed rats (Fig. 3f, g), suggesting that EFV did not affect anxiety. Finally, no differences were found in the total distance moved (*F*_(1, 41)_ = 0.86, *p* = 0.361; Fig. 3h), indicating that EFV had also no effect on exploratory locomotion. Due to video recording errors, swimming scores could not be determined for two EFV-exposed rats. While there were no differences between the groups in the total duration of immobility (*F*_(1, 39)_ = 0.64, *p* = 0.48), or the total number of immobility counts (*F*_(1, 39)_ = 0.598, *p* = 0.44), there was a significant difference in latency to the first episode of immobility (*F*_(1, 39)_ = 9.88, *p* = 0.003), with EFV-exposed rats exhibiting reduced latencies (Fig. 3i–k).

### Motor cortex cytoarchitecture

**Fig. 4:**
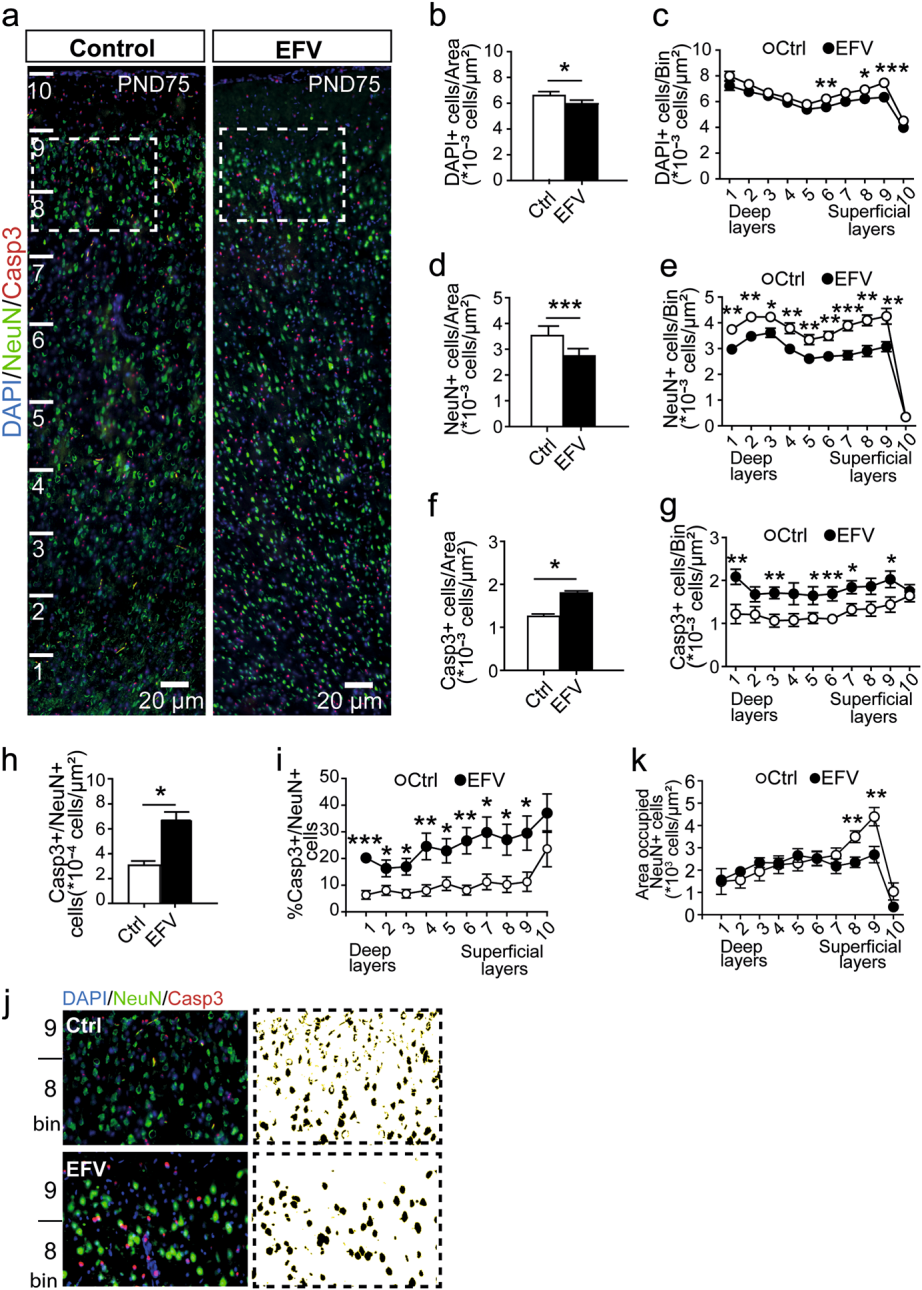
Perinatal exposure of EFV increases apoptosis in neurons of the motor cortex (M1)**.** **a** Immunostaining for DAPI, mature neurons (NeuN) and apoptosis (Caspase-3; Casp3) in the primary motor cortex (M1) of EFV-exposed (*n* = 8) and Ctrl-exposed (*n* = 8) animals. The boxed areas are enlarged in (**j**). **b**, **c** Significant decrease of the total number of DAPI^+^ cells in the superficial cortical layer of the EFV-exposed group. **d**, **e** Quantification of mature neurons (Neun^+^) showing a significant decrease in Neun^+^ cells in deep and superficial layers in the EFV-exposed group. **f**, **g** Quantitative results showing a significant increase in Casp3^+^ cells in deep and superficial layers of the M1 of EFV-exposed animals. **h**, **i** A significant increase in Casp3^+^/Neun^+^ cells in the EFV group compared with controls. **k** Significant differences in the area occupied by Neun^+^ nuclei in two bins of superficial layers between EFV-treated and control animals. Data show mean ( ± SEM) number of cells, *p < 0.05, **p < 0.01, ***p < 0.001, analyzed using one-way ANOVA. Ctrl vehicle, EFV efavirenz, ANOVA analysis of variance

To investigate whether perinatal EFV exposure affected the architecture of the primary motor cortex (M1), we assessed the M1 cell composition in randomly selected brains from adult animals (PND73–75; Fig. 4a). We observed a reduction in the total number of DAPI^+^ cells in the M1 of the EFV-exposed compared with the control group (*p* = 0.045; Fig. 4a–c and Supplemental Figure S1b). This decrease was present in the superficial layers (bin8, *p* = 0.014; bin9, *p* = 0.00030) and to a lesser extent in deep layers (bin6, *p* = 0.0096). Groups did not differ, however, in M1 cortical thickness (*p* = 0.37; Supplemental Figure S1g). To assess whether the observed reduction in cells was neuron specific, we immunostained the M1 for the neuronal nuclei marker NeuN (Fig. 4a, d, e). We found a significant reduction of NeuN^+^ neurons in the M1 of the EFV-exposed group (*p* < 0.0001; Fig. 4d and Supplemental Figure S1c), which was present in all layers (bin1–bin9, *p* < 0.05; Fig. 4e). Next, we focused on cell apoptotic features of perinatal EFV exposure in the M1 (Fig. 4a, f, g). We observed a significant increase of Casp3^+^ cells in the M1 of EFV-exposed animals (*p* = 0.023; Fig. 4f and Supplemental Figure S1d), which was apparent in deep (bin1, *p* = 0.0087; bin3, *p* = 0.0059; bin5, *p* = 0.049 and bin6, *p* = 0.0071) and superficial layers of the M1 (bin7, *p* = 0.023 and bin9, *p* = 0.041; Fig. 4g) of the M1. The number of NeuN^+^ neurons positive for Casp3 was higher in the EFV-exposed group compared with the control group (*p* = 0.037; Fig. 4h, i and Supplemental Figure S1e,f). In addition, we noticed a small population of Casp3^+^ cells that were astrocytes (positive for GFAP) (Supplemental Figure S1a). When comparing nuclear size (using NeuN), we observed significantly reduced nuclear sizes in superficial layers of the EFV-exposed group (bin8, *p* = 0.0054; bin9, *p* = 0.0080; Fig. 4j, k), suggesting a possible difference in apoptotic state. Together, these data suggest (neuronal) cell loss in M1 cortical areas, which persists into adulthood in perinatally EFV-exposed animals.

**Fig. 5:**
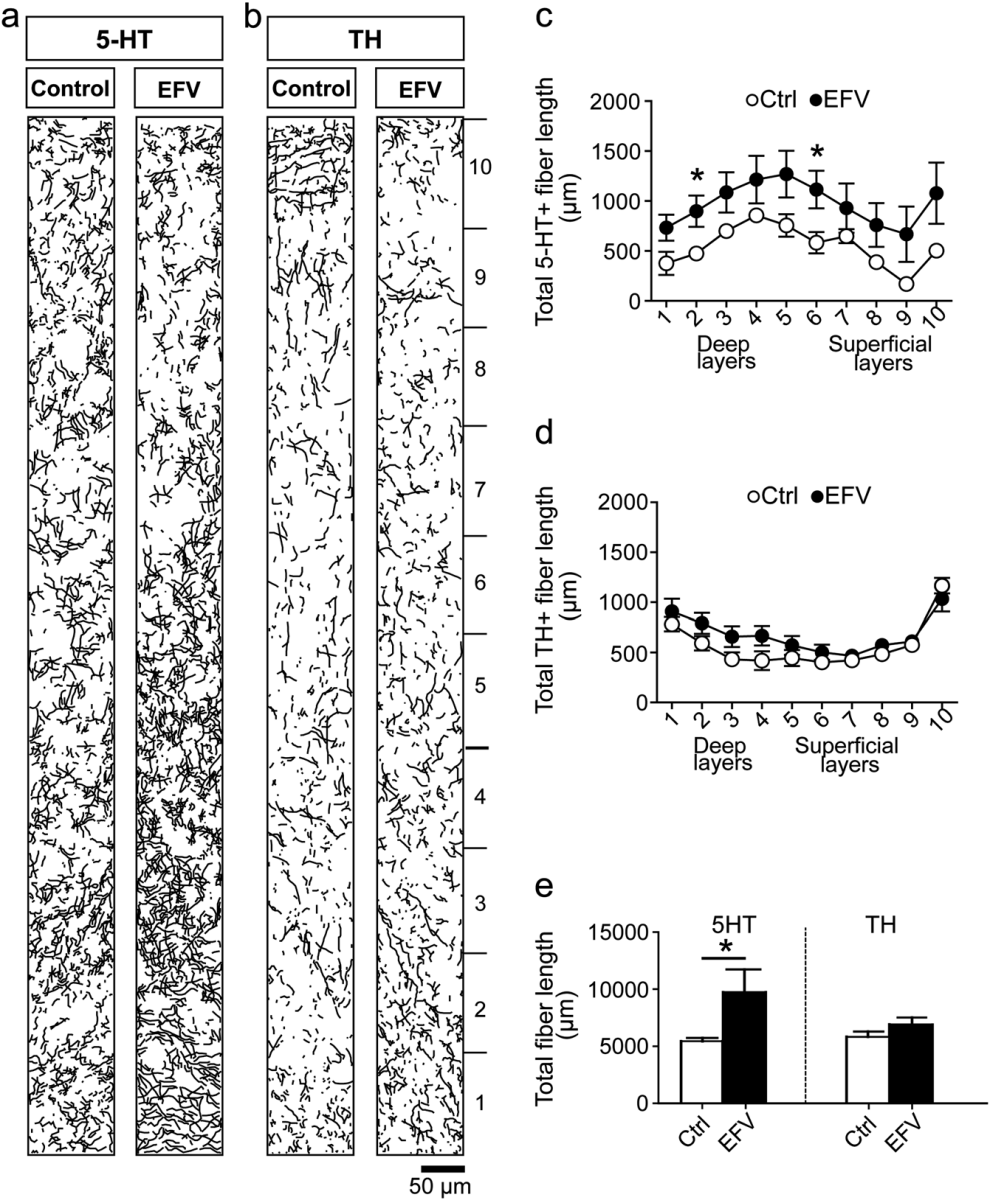
Perinatal exposure to EFV affects serotonergic (but not catecholaminergic) innervation of the M1. **a**, **b** Camera lucida drawings of immunostaining for **a** 5-HT^+^ and **b** TH^+^ fibers in deep and superficial layers of the M1 in EFV-exposed and Ctrl-exposed brains (*n* = 7 per group for TH^+^ and *n* = 5 per group for 5-HT^+^). **c** Significant increase in 5-HT^+^ fiber length in bin2 and 6 in the EFV group. **d**, **e** Significant increase in 5-HT^+^ fiber length, but no difference in TH^+^ fiber length in the total M1 area of the EFV-exposed group. Data show mean ( ± SEM) number of fibers, *p < 0.05 analyzed using one-way ANOVA. 5-HT serotonin, Ctrl vehicle, EFV efavirenz, TH tyrosine hydroxylase, ANOVA analysis of variance

Given the serotonergic pharmacological profile of EFV, we hypothesized that early EFV exposure could have long-term effects on the serotonergic system and potentially indirectly on other neurotransmitter systems such as the catecholaminergic system^30^. To test this hypothesis, we immunostained M1 cortical slices for 5-HT and TH (Fig. 5a, b) and found that perinatal EFV exposure was associated with increased 5-HT^+^ fiber length (*p* = 0.028; Fig. 5c, e) in deep and superficial layers of the M1 (bin2, *p* = 0.034; bin6, *p* = 0.040; Fig. 5c). We found no differences in TH^+^ fiber length (*p* = 0.19; Fig. 5d, e), suggesting that perinatal exposure to EFV influences the development and maintenance of the serotonergic, but not the catecholaminergic, system.

### Correlations between reflex and motor development and M1 cytoarchitecture

Swimming performance correlated positively with the number of cells (DAPI^+^; *r*_s_ = 0.631, *p* = 0.009, Supplemental Figure S2) and post-mitotic neurons (NeuN^+^; *r*_s_ = 0.642, *p* = 0.007) in the M1. In addition, we found a significant positive correlation between negative geotaxis and 5-HT^+^ fiber length (*r*_s_ = 0.700, *p* = 0.036) and a trend between negative geotaxis and the number of DAPI^+^ cells in M1 (*r*_s_ = −0.492, *p* = 0.053). These results suggest that the M1 of animals that performed better on the motor-related tests contained more DAPI^+^ cells, more neurons and shorter 5-HT fibers.

## Discussion

In this study, we demonstrate that perinatal exposure to EFV in rats results in a transient delay in reflex and motor development and in persistent changes in M1 cytoarchitecture. Since maternal care was not affected by EFV treatment, the behavioral changes most likely arise from direct EFV brain toxicity. Correspondingly, EFV exposure caused persistent structural changes in motor cortical layers, reflected by a reduction in the number of mature neurons. The increased number of Casp3^+^ neurons points to neuronal cell death as the underlying cause. Finally, we found increased serotonergic, but not catecholaminergic, innervation of the M1 in EFV-exposed rats compared with controls, indicating that EFV, either directly or indirectly, affects the serotonergic system.

At the outset of our study, no studies had been published on long-term neurodevelopmental effects of EFV. Given EFV’ ability to interfere with the brain serotonergic system^12–14^, we focused on behavioral domains known to be affected by genetic or pharmacological inactivation of the 5-HTT: reflex development^22,40^, motor performance^21,25^, and emotion^24,41^. We found that perinatal EFV exposure resulted in delayed reflex development, reflected by increased latencies in the negative geotaxis test, and reduced startle responses. The reduced startle responses may indicate decreased capability of these rats to react to new auditory stimuli, but also point to a reduced ability to initiate motor responses. Correspondingly, EFV-exposed rats performed poorer in the swimming and bar holding tests. Adequate swimming requires the smooth integrative organization of multiple reflexes, including vestibular reflexes and extensor-flexor reflexes, which progressively develop during the postnatal phase of CNS maturation^35^. The delayed development of swimming ability in EFV-exposed rats suggests that early EFV exposure might modify these integrated neuromuscular mechanisms. The fact that bar holding performance was reduced, as well indicates that muscle strength may have contributed to reduced swimming performance. We observed no group differences in PPI, the elevated plus maze test, or behavioral despair in the forced swim test. We did, however, observe shorter latencies to immobility in the forced swim test, possibly reflecting motor-related problems. Taken together, these results suggest that perinatal exposure to EFV affects reflex and motor development, but not emotional behavior. The results are in line with recent findings showing that, compared to HIV-unexposed infants, HIV-uninfected infants perinatally exposed to EFV (aged 12–14 months) are at an increased risk for motor delay (particularly those born prematurely)^6^.

Alterations in care provided by the mother can potentially mediate effects of EFV on motor development. As one-third of EFV-treated adults report CNS symptoms^10,42^, and EFV-treated adult rats show anxiety and depressive-like behavior^14,43^, dams treated with EFV may have provided lower-quality care of pups than mothers treated with vehicle. For this reason, we measured maternal care and did not find significant differences between EFV- and vehicle-treated dams. Hence, the observed behavioral and morphological neurodevelopmental changes in EFV-exposed rats likely reflect (in)direct drug toxicity, rather than indirect effects mediated by altered maternal care.

To elucidate to what extent perinatal drug toxicity effects could explain the observed delay in reflex- and motor development, we investigated the cytoarchitecture of the motor cortex (region M1) in adult rats. In the M1 of perinatally EFV-exposed rats, we observed a marked decrease in total cell numbers, including mature neurons, and an increase in the number of neurons that expressed the apoptotic marker Casp3. These findings indicate that perinatal EFV causes cell death later in life, leading to a partial loss of neurons. The positive correlation between motor development and number of cells suggests that the delayed motor function is related to reduced cell number in the M1. The EFV-induced loss of cells may be attributed to EFV’ (in)direct ability to interfere with cellular metabolism. For example, EFV has been shown to decrease neuronal ATP storage, leading to increased levels of reactive oxygen species and cell death^44,45^.

Alternatively, perinatal EFV exposure could indirectly lead to neurodevelopmental changes and cell death, by targeting the serotonergic system. We therefore determined whether the observed neuronal loss and cell death were related to changes in the serotonergic system. We measured the length of 5-HT^+^ and TH^+^ fibers in the M1 and observed an increased length of 5-HT^+^, but not TH^+^, fibers, which correlated with reduced performance in the negative geotaxis test. This result resembles the case of perinatal SSRI exposure, which also results in increased cortical serotonergic innervation in later life^19,46^. 5-HT is one of the earliest neurotransmitter in the brain and performs neurotrophic functions during early development^47^. Hence, any interference with the serotonergic system by perinatal drug exposure can have profound long-lasting indirect effects on neurodevelopmental events. The 5-HT2, 5-HT3, and 5-HT6 receptors are involved in cell division^48^, differentiation^49^, survival^50^, and neuronal migration pathways^47^. EFV, as a 5-HT receptor (ant)agonist^13^, could directly interfere with these processes, consequently leading to neuronal cell death^45^. Elucidation of the exact molecular and cellular consequences at various developmental time points after perinatal EFV exposure in cortical and other brain areas requires further research.

We observed no signs of EFV-related pregnancy complications or teratogenicity, which is in line with findings in a recent meta-analyses including 2026 first-trimester EFV exposures^5^. Still, EFV-exposed rats showed delayed maturation (delayed eyelid opening) and reduced body weights throughout life. Reduced birth weights have been reported in EFV-exposed infants, although most studies in humans report comparable birth weights with infants exposed to other ART^4,51,52^. Interpretation of these data is difficult as drug-related effects cannot easily be isolated from other HIV-related factors. Interestingly, the reduced body weight finding is in line with animal and human data derived from other early-life 5-HT stimulation models^24,25,53^. There are several ways in which EFV exposure might reduce body weight: by 5-HT-dependent mechanisms, through interference with hypothalamic 5-HT receptors and central regulation of eating behavior^54^, or by 5-HT-independent mechanisms such as increased metabolism and altered adipocyte differentiation^55^.

In contrast to other behavioral EFV studies^14,43^, we measured plasma levels of EFV. Using a 100 mg/kg daily dose, we produced EFV plasma levels that were detectable, yet below the therapeutic range in humans (1.0–4.0 mg/L)^56^. One could argue that our experiment was not representative for the human situation. However, it is known that the main neurotoxic metabolite of EFV approximates human exposure, despite apparent relatively low EFV plasma levels^55^, In addition, even at relatively low concentrations, EFV has been shown to extensively accumulate in brain tissue in rats^57^. Finally, at similar doses in rats, EFV has been shown to induce neurotoxic changes^12^. We, therefore, postulate that our experimental set-up was sufficiently valid to induce the desired effects.

The major strength of using a rat model is that potential HIV-related and socioeconomic confounders of adverse neurodevelopmental effects of EFV in humans (e.g., parental loss; co-medication) can be eliminated. As EFV did not affect maternal care, developmental effects through care provided by the mother can be excluded. Our study also has limitations. We performed multiple behavioral tests with the same animals, which could have influenced test outcomes. We expect this carry-over effect to be limited, as tests were generally not stressful. Furthermore, behavioral and molecular experiments were performed with the same animals. Motor behavior was measured mostly during early life, while we only have molecular data from adult rats. This makes it difficult to draw detailed mechanistic conclusions The significant correlations between adult M1 cortical architecture and early motor performance, however, suggest that the M1 molecular changes arise early during development and persist throughout adulthood. Future studies using multiple time points and, if possible, interventions, are needed to study underlying mechanisms into more detail.

There is a clear indication of HIV treatment during pregnancy as it not only protects the (unborn) child, but also benefits the mother’s health. The vast majority of children exposed to EFV during pregnancy will be HIV uninfected with normal life expectancies. However, this outcome may come at a cost. As we demonstrate, perinatal exposure to EFV in rats leads to a transient delay in reflex and motor development, and a long-lasting loss of neurons in the motor cortex. Thus, EFV could affect the development of children who may already be experiencing multiple adverse conditions (such as having a mother living with HIV). Our findings underline the need for long-term clinical studies in children that are perinatally exposed to EFV, as well as more detailed studies on the underlying neurodevelopmental mechanisms.

## Data deposition

Data used in this article are available from the corresponding authors upon request.

## Supplementary information

Supplementary Information.
