# Supplementary material for: Neurodevelopmental and behavioral consequences of perinatal exposure to the HIV drug efavirenz in a rodent model
Source: Transl Psychiatry. 2019 Feb 11;9:84. doi: 10.1038/s41398-019-0420-y (PMC6370772; doi:10.1038/s41398-019-0420-y)
Supplement: Supplementary file 1 — Supplementary Information. [file 41398_2019_420_MOESM1_ESM.docx]

**Supplementary Information**

**Title** Neurodevelopmental and behavioral consequences of perinatal exposure to the HIV-drug efavirenz in a rodent model

Lisa Van de Wijer, MSc,^1,7^ Lidiane P. Garcia, MSc, ^2,7^ Sabrina I. Hanswijk, MSc,^3^ Juliette Rando, BSc;^3^ Anthonieke Middelman, MSc;^3^ Rob ter Heine, PhD,^4^ Quirijn de Mast, PhD^1^, Gerard J.M. Martens, PhD^2^, André J.A.M. van der Ven, PhD^1^, Sharon M. Kolk, PhD^2,6,8^, Arnt F.A. Schellekens, PhD^5,8^, Judith R. Homberg, PhD^3,8^

*^1^Department of general internal medicine, Radboud university medical center, Nijmegen, The Netherlands*

*^2^Department of Molecular Animal Physiology, Donders Institute for Brain, Cognition, and Behaviour, Centre for Neuroscience, Radboud University, Nijmegen, The Netherlands*

*^3^Department of Cognitive Neuroscience, Donders Institute for Brain, Cognition, and Behaviour, Centre for Neuroscience, Radboud university medical center, Nijmegen, The Netherlands*

*^4^Department of Pharmacy, Radboud university medical center, Nijmegen, The Netherlands.*

*^5^Department of Psychiatry, Donders Institute for Brain, Cognition, and Behaviour, Centre for Neuroscience, Radboud university medical center, Nijmegen, The Netherlands*

*^6^Department of Molecular Neurobiology, Donders Institute for Brain, Cognition, and Behaviour, Centre for Neuroscience, Radboud University, Nijmegen, The Netherlands,*

*^7^Authors contributed equally to this work*

*^8^Authors contributed equally to this work*

**Judith Homberg, PhD**

Department of Cognitive Neuroscience

*Donders Institute for Brain, Cognition and Behaviour, Centre for Neuroscience*

Radboudumc

Kapittelweg 29

6525 EN Nijmegen

The Netherlands.

[judith.homberg@radboudumc.nl](mailto:judith.homberg@radboudumc.nl)

Phone: +31-(0)24-3610906

**Arnt F.A. Schellekens, PhD**

Department of Psychiatry

*Donders Institute for Brain, Cognition, and Behaviour, Centre for Neuroscience*

Radboudumc

Reinier Postlaan 10

6525 GC Nijmegen

The Netherlands

[arnt.schellekens@radboudumc.nl](mailto:arnt.schellekens@radboudumc.nl)

Phone: +31-(0)243613400

**Sharon M. Kolk, PhD**

Department of Molecular Neurobiology

*Donders Institute for Brain, Cognition, and Behaviour, Centre for Neuroscience*

Radboud University

Heyendaalseweg 135
6525 AJ Nijmegen
The Netherlands

[S.Kolk@donders.ru.nl](mailto:S.Kolk@ncmls.ru.nl)
Phone: +31-(0)24-3610565

***Supplemental Table 1. Animal numbers used for each test***

| **Test** | **Efavirenz**  **No.** | **Control**  **No.** |
| --- | --- | --- |
| Behavioral tests |  |  |
| Body weight | 24 | 19 |
| Eye opening | 24 | 19 |
| Righting reflex | 24 | 19 |
| Negative geotaxis | 24 | 19 |
| Startle reflex^*^ | 23 | 16 |
| PPI^*^ | 20 | 16-17 |
| EPM | 24 | 19 |
| Forced swim test*^†^* | 22 | 19 |
|  |  |  |
| Immunohistochemistry*^‡^* |  |  |
| DAPI+, NeuN, Casp3 | 8 | 8 |
| TH | 7 | 7 |
| 5-HT | 5 | 5 |

*5-HT serotonin; Casp3 caspase 3; EPM elevated plus maze; NeuN neuronal nuclei marker; PPI prepulse inhibition; TH tyrosine hydroxylase.*

*^*^Extreme outliers excluded from analyses according to Tukey’s principles. ^†^Due to video-recording error swimming scores could not be determined for two efavirenz-exposed rats.*

***Supplemental Table 2. General litter characteristics***

| **Test** | **Efavirenz**  **No.=4** | **Control**  **No.=4** | **P-value** |
| --- | --- | --- | --- |
| Gestational length, days | 22 (0.2) | 22 (0.0) | 0.69 |
| No. of pups per litter | 10.8 (0.7) | 8.8 (1.3) | 0.34 |
| No. of still births | 0 (0.0) | 0 (0.0) | - |
| No. of neonatal deaths | 0.5 (0.3) | 0.0 (0.0) | 0.34 |
| Gender pups, % female | 42 (7) | 41 (12) | 0.89 |
| Efavirenz plasma level (mg/L) | 0.28 (0.13) | - | - |

*Data are depicted as mean (±SEM)*

***
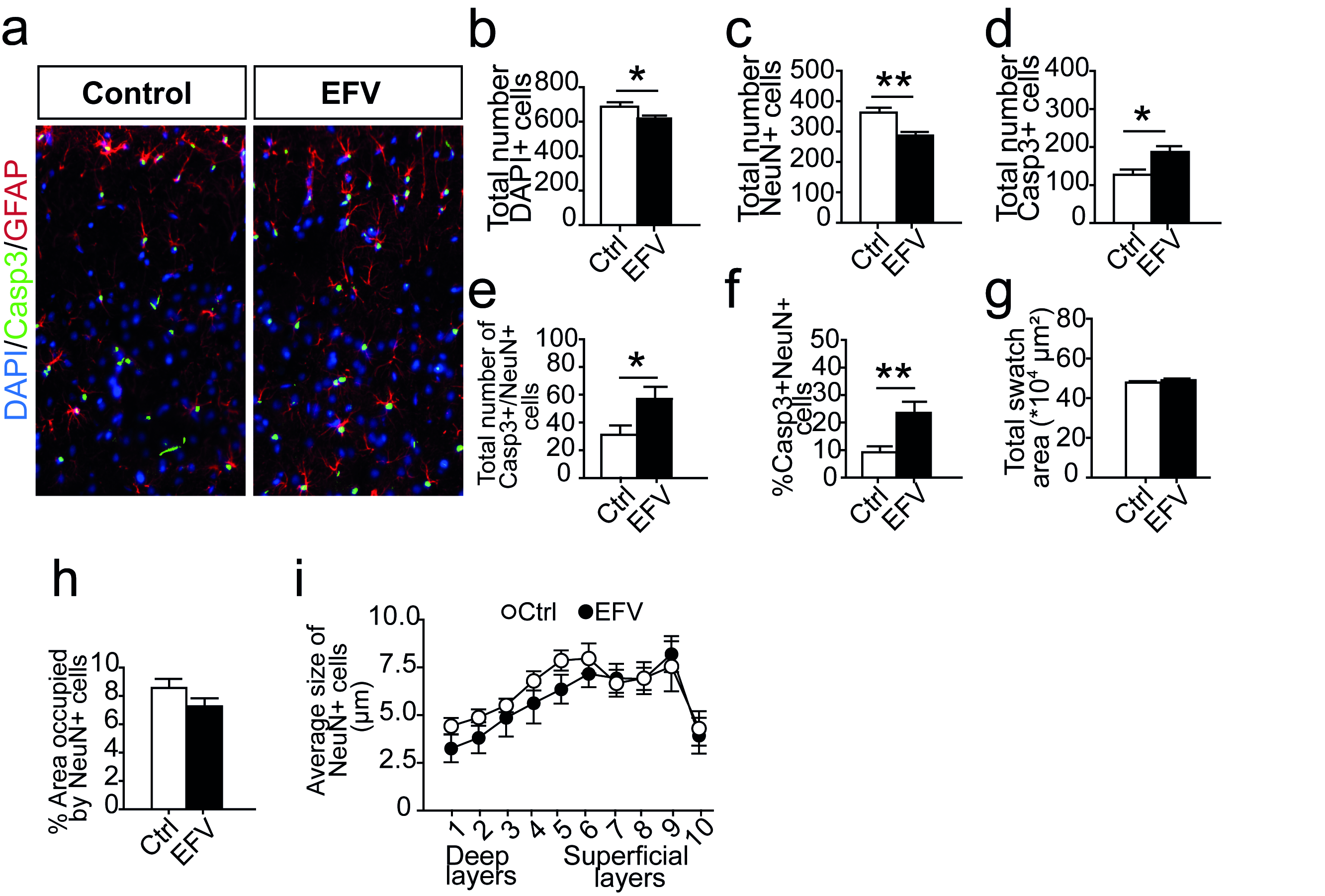
***

***Supplemental Figure S1. Perinatal exposure of EFV increases apoptosis in several types of motor cortex cells including astrocytes (a-f), but has no effect on motor cortical thickness.*** *Ctrl vehicle ; EFV efavirenz (****a****) Swatch of immunostaining showing colocalization with Caspase3 (Casp3) and GFAP, an astrocyte marker, in the motor cortex of EFV- (n=8) and Ctrl-exposed (n=8) animals. (****b,c,d****) Significant decrease in the total number of cells (****b****), total number of mature neurons (****c****) and total number of cells that are Casp3^+^ (****d****) in the EFV-exposed group compared with the Ctrl-exposed group. (****e****) Significant increase in the total number of Casp3^+^ neurons. (****f****) Increased percentage of neurons that are Casp3^+^ in the EFV-exposure group. (****g****) No differences in the total length of a cortical swatch (****h,i****) No differences in the percentage of area occupied by NeuN^+^ cells and average size of NeuN^+^ cells in EFV group compared to the control group. Data show average ± SEM. One-Way ANOVA, *p<0.05, **p<0.01.*

***
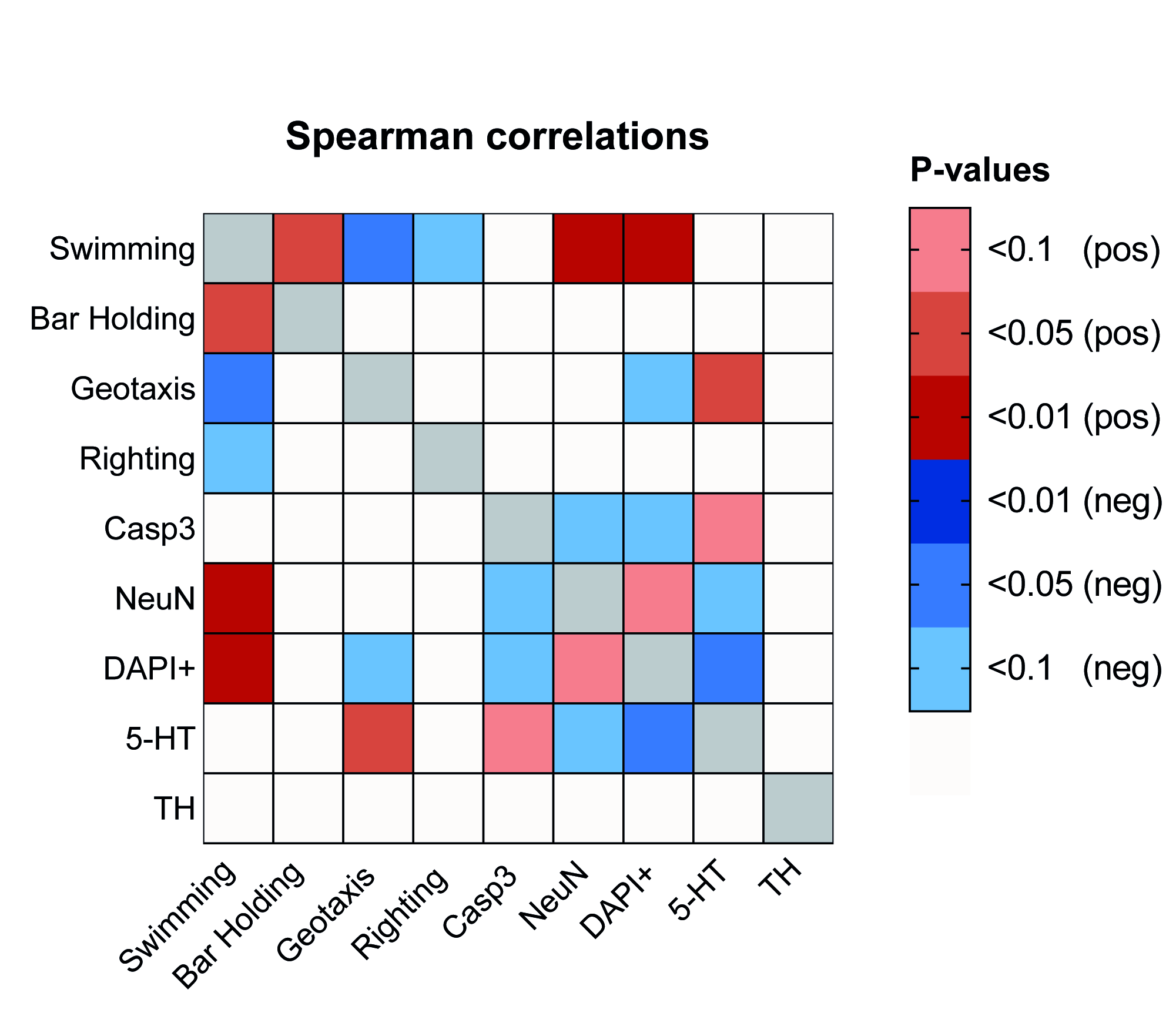
***

***Supplemental Figure. S2. Spearman correlations between behavioral development and immunohistochemistry data of control (n=8) and efavirenz-exposed (n=8) animals.***

*Cumulative behavioral development was defined using area-under-the-curve for righting reflex (PND2-10), negative geotaxis (PND4-14) and bar holding (PND10-21) and total sum score for swimming performance (PND 8,10,12,14,22). Immunohistochemistry data reflect the total number of DAPI^+^ cells, mature neurons (NeuN^+^), apoptotic cells (caspase3 (Casp3)), total serotonin (5-HT^+^) fiber length, and total TH^+^ fiber length in the primary motor cortex (M1).*
